# Novel Alternative Block for Scoliosis Surgery: Serratus Posterior Superior Plane Block, Does Not Need to Visualize the Transverse Process

**DOI:** 10.5152/eurasianjmed.2024.23346

**Published:** 2024-10-01

**Authors:** Bahadir Ciftci, Selcuk Alver, Burak Omur, Ali Ahiskalioglu

**Affiliations:** 1 Department of Anesthesiology and Reanimation, Istanbul Medipol University School of Medicine, Istanbul, Türkiye; 2 Department of Anesthesiology and Reanimation, Ataturk University School of Medicine, Development and Design Application and Research Center, Erzurum, Türkiye

To the Editor,

Idiopathic scoliosis is a common deformity in the pediatric population.^1^ Effective postoperative analgesia management prevents complications such as atelectasis and pneumonia. Erector spinae plane block (ESPB) is performed between the transverse process (TP) and erector spinae muscle. Erector spinae plane block may be performed for thoracolumbar surgeries, and it provides effective analgesia management. However, performing ESPB after scoliosis surgery has some disadvantages. The anatomical structure of muscles and bones changes after the surgery due to the nails and instruments used for surgery, and often it becomes very difficult to visualize the TP due to surgery.^2^ Mislovic et al have performed ESPB laterally over the rib instead of a TP.^2^ They reported that since they could not visualize the TP due to anatomical changes caused by the previous spinal surgery, they performed ESPB above the thoracic rib. However, the spread of local anesthetic should be seen in the deep part of the erector spinae muscle over the TP.^3^ The Serratus posterior superior intercostal plane block (SPSIPB) is a novel block that has been defined by Tulgar et al.^4^ Serratus posterior superior intercostal plane block is performed between the SPS muscle (SPSm) and the third rib, and it targets intercostal nerves. Serratus posterior superior intercostal plane block provides a sensorial block from C3 to T10 levels.^4^ Since the anatomical landmark is the rib, SPSIPB may be an alternative regional technique in patients with difficult-to-see TP due to spinal surgery. We want to report our SPSIPB experience with a pediatric patient who underwent scoliosis surgery.

Written informed consent was obtained from the patient’s parent for this report. A 10-year-old, 50 kg, 140 cm female patient underwent scoliosis surgery. Posterior instrumentation between T2 and L1 and thoracic scoliosis stabilization surgeries were performed. We administered a dose of 500 mg paracetamol and 50 mg tramadol IV before the end of the surgery. We aimed to perform ESPB for postoperative analgesia management. Under sterile conditions, we scanned from the midline with a linear high-frequency transducer (11–12 MHz) in the median sagittal plane; however, we could not identify the TP on both surgical sides. We tried to perform ESPB on this patient at several levels. We scanned the TP from T2 to T10 with ultrasound but could not see it. Thus, we decided to perform SPSIPB. The third rib was visualized near the medial border of the scapula. A 22 G, 50 mm block needle was inserted between the rib and the SPSm. Afterward, 10 mL of 0.25% bupivacaine was administered bilaterally (a total of 20 mL). We administered 500 mg of paracetamol IV every 8 hours postoperatively. During the postoperative period, the numerical rating score was <3, and there was no need for additional analgesic drugs or opioid agents (Table 1).

Serratus posterior superior intercostal plane block provided effective postoperative analgesia management for our pediatric patient. Since SPSIPB is more lateral from the midline and the anatomical landmark is the rib, it may be a better choice than ESPB for spine surgery. The spread of SPSIPB is between the C3 and T10 levels. However, in this case, the surgical level extends to L1. We applied 10 mL of local anesthetic to this pediatric patient. Local anesthetic spread may be different in pediatric patients due to anatomical differences. Since the muscles are thinner and the fascial planes are more flexible, the local anesthetic spread may be wider in pediatric patients. We also applied a multimodal analgesia protocol to our patient. Therefore, we believe that effective analgesia occurred in our patient. The SPSPB is a regional anesthetic technique primarily used to provide analgesia to the chest wall. This block targets the fascial plane of the SPSm, which is located deep in the trapezius and rhomboid muscles. The mechanism of action involves the injection of a local anesthetic into the fascial plane below the SPSm, where it diffuses to block the intercostal nerves and dorsal rami that supply the thoracic area. This results in reduced sensation and pain transmission from the affected dermatome levels, providing effective pain relief for conditions like rib fractures, thoracic surgery, or chronic chest pain syndromes. Analgesic techniques for scoliosis surgery are crucial for effective pain management, allowing for better postoperative recovery and rehabilitation. Common methods include intravenous opioids, epidural analgesia, and paravertebral blocks. Each technique has its benefits and potential drawbacks; for instance, opioids can lead to side effects such as nausea, constipation, and respiratory depression, while epidural analgesia, although effective, can cause hypotension, urinary retention, and, in rare cases, neurological complications.^1^ Paravertebral blocks offer a more targeted approach but can be technically challenging and carry risks, such as pneumothorax. The serratus posterior superior intercostal plane block presents a potential advantage over these methods by providing localized analgesia to the thoracic area without significantly affecting motor function or causing the systemic side effects associated with opioids. Additionally, it is generally easier to administer than paravertebral blocks and avoids the complications associated with epidural techniques, making it a safer and more patient-friendly option in the perioperative management of scoliosis surgery.

As a limitation, we did not evaluate the sensory blockade of the patient. Cadaveric investigations and radiologic imaging studies are needed to explain the exact spread of SPSIPB. SPSIPB may be a good alternative analgesic method for scoliosis surgery.

## Data Availability Statement

The data is available on reasonable request from the corresponding author.

## Figures and Tables

**Table 1.** The demographic data and pain scores of the patient

Patient	Gender	Age	Height/Weight	NRS First Hour	NRS Fourth Hour	NRS Eighth Hour	NRS 16th Hour	NRS 24th Hour
1	Female	10	140 cm, 50 kg	2	1	0	0	1

NRS, Numerical rating score.
